# Blood Lead Levels and Subsequence Risk of Malaria in the African Population: A Systematic Review and Meta-Analysis

**DOI:** 10.3390/tropicalmed6030149

**Published:** 2021-08-08

**Authors:** Saruda Kuraeiad, Manas Kotepui

**Affiliations:** Department of Medical Technology, School of Allied Health Sciences, Walailak University, Tha Sala, Nakhon Si Thammarat 80160, Thailand; saruda.ku@wu.ac.th

**Keywords:** malaria, BLL, *Plasmodium*, severity, lead toxicity

## Abstract

Previous epidemiological studies showed that blood lead level (BLL) was associated with malaria infection and severity. Therefore, the present study aimed to qualitatively and quantitatively synthesize the evidence on the association between BLL and risk of malaria infection and severity using the systematic review and meta-analysis approach. Potentially relevant studies were identified from three databases using a combination of search terms. The quality of the included studies was assessed using the checklist for the cross-sectional studies developed by the Joanna Briggs Institute. The qualitative synthesis of the risk or odds of malaria infection in patients with BLL was performed as the outcome of each included study could not be pooled. The pooled mean BLL and prevalence of malaria infection of the included studies was estimated using a random-effect model. The heterogeneity of the outcomes among the included studies was assessed using the Cochran Q test and I^2^ statistics. The subgroup analysis of the study sites and participants was performed to explore the source(s) of heterogeneity of the outcomes. Publication bias was assessed in the case of more than 10 studies used for pooling of the same outcome. Among 114 potentially relevant studies identified from the databases, 6 eligible studies were included for qualitative and quantitative syntheses. The results showed that the pooled mean BLLs were 7.33 μg/dL in children (95% confidence interval (95%CI), 4.08–10.58; I^2^, 98.2%), 7.94 μg/dL in children with BLL > 45 mg/dL before chelation (95%CI, 7.87–8.01), 7.41 μg/dL in infants (95%CI, 7.34–7.48 μg/dL), 9.20 μg/dL in children with malaria (95%CI, 9.16–9.24 μg/ dL), and 36.37 μg/dL in pregnant women (95%CI, 34.43–38.31 μg/dL). The prevalence rates of malaria among participants (2381 participants, 803 malaria-positive patients) were 53% in children (95%CI, 50–57%; I^2^, 99.8%), 24% in children with BLL > 45 mg/dL before chelation (95%CI, 21–27%), 12% in infants (95%CI, 8–18%), and 21% in pregnant women (95%CI, 18–26%). The subgroup analysis of countries demonstrated that the prevalence rates of malaria among participants was 17% in Benin (95%CI, 13–21%; I^2^, 98.8%) and 36% in Nigeria (95%CI, 10–63%; I^2^, 99.4%). BLL associated with decreased risk of malaria was demonstrated by two studies conducted in Benin and Nigeria, while BLL associated with increased risk of malaria was demonstrated by a study conducted in Nigeria. BLL was associated with the risk of severe malaria, involving severe neurological features and severe anemia. In conclusion, the present systematic review and meta-analysis determined the current status of the studies on BLL and risk of malaria in African countries. Further studies are needed to investigate the impact of BLL on patients with malaria to help the clinician determine the risk of severity, such as the development of neurological features or severe anemia, among patients exposed to lead.

## 1. Background

Lead is a heavy metal extensively found in paint, gasoline, electronic waste, batteries, glazed ceramics, lead-contaminated utensils, lead-contaminated water, cosmetics, traditional medicines, industrial emissions, older homes, dust, imported toys, mining and smelting industries, and spices, and there is also the industrial legacy of lead exposure and batteries [1,2,3]. Lead continues to be a public health problem for individuals with short- and long-term exposures. The long-term effects of lead exposure are unclear. However, several epidemiological studies and systematic reviews showed the association between lead exposure and several diseases or disorders, including cognitive impairment [4], cardiovascular disease [5,6], renal disease [7], decreased fertility [8], neurotoxicity [9], cancer [10], and malaria [11].

Malaria is caused by the infection of the protozoa genus *Plasmodium*. Recently, eight *Plasmodium* species were reported to cause naturally acquired infections in humans, including *Plasmodium falciparum*, *Plasmodium vivax*, *Plasmodium ovale curtisi*, *Plasmodium ovale wallikeri*, *Plasmodium knowlesi*, *Plasmodium brasilianum*, and *Plasmodium cynomolgi* [12,13,14]. Among these eight species, *Plasmodium*, *P*. *falciparum*, *P*. *knowlesi*, and some *P*. *vivax* infections were known to cause severe malaria [15,16]. Recently, the World Health Organization (WHO) estimated that 229 million malaria cases and 409,000 deaths were reported in 2019, of which 94% of malaria cases and deaths were recorded in African countries [17].

Previous epidemiological studies showed that blood lead level (BLL) was associated with malaria infection [11,18,19]. Moreover, some studies reported that BLL increased the risk of severe malaria, including that of neurological disorder [20] and severe anemia [21]. However, limited data were available to verify the evidence. Therefore, the present study aimed to qualitatively and quantitatively synthesize the evidence of the association between BLL and risk of malaria infection and severity using the systematic review and meta-analysis approach. The results of this study will provide information on the current status and provide evidence of BLL and malaria infection for further studies.

## 2. Methods

### 2.1. Protocol and Search Strategy

The protocol of this systematic review was registered at PROSPERO (ID CRD42021260017). The reports of the systematic review complied with the Preferred Reporting Items for Systematic Reviews and Meta-analyses (PRISMA) [22]. The search strategy involved the combination of search terms “(‘lead exposure’ OR ‘lead toxicity’ OR ‘blood lead’ OR ‘lead level’ OR ‘lead pollution’ OR ‘exposure to lead’ OR ‘lead level’ OR ‘lead poisoning’ OR ‘lead ions’) AND (malaria OR plasmodium)” as provided in Supplementary Materials, Table S1. The searches were performed in PubMed, Web of Science, and Scopus. The search was not limited by language or publication date and was completed on 20 May 2021. 

### 2.2. Eligibility Criteria

Cross-sectional studies that reported the BLL of patients with malaria were reviewed and included if eligible. The following studies were excluded: experimental studies, animal studies, reviews, systematic reviews, comments, letters to the editor, case reports, and case series.

### 2.3. Study Selection and Data Extraction

Study selections were performed by authors based on the eligibility criteria. Any disagreement on study selection between two authors was discussed for consensus. First, the potentially relevant studies were identified from three databases; then, duplicates were removed. Second, the titles and abstracts of the remaining studies were screened; then, unrelated studies were excluded. Third, the remaining studies were examined for full texts; then, unrelated studies were excluded with reasons. Finally, the eligible studies were included for further review and meta-analysis. Data of the included studies were independently extracted into the pilot standardized datasheet by two authors. The following data were extracted from each study: author, year of publication, study site, year of conducted the study, study design, number of participants, characteristics of the participants, age range and mean age, sex, research questions, mean and standard deviation of BLL, number of malaria cases, outcome, outcome parameters, diagnostic tests for malaria, and method for BLL assessment.

### 2.4. Quality of the Included Studies

The quality of the included studies was assessed using the checklist for cross-sectional studies developed by the Joanna Briggs Institute [23]. The checklist was based on the following eight criteria: inclusion criteria, details of study subjects and setting, measurement of exposure, standard criteria used for measurement of the condition, identification of confounding factors, strategies to deal with confounding factors, measurement of outcomes, and appropriateness of statistical analysis. Any study that met at least seven criteria indicated a high-quality study, while any study that met four to six criteria indicated a moderate quality. Any study that met less than four criteria indicated a low quality and was then excluded from the present study.

### 2.5. Data Syntheses

Data syntheses were divided into two parts. The first part was the qualitative synthesis of the evidence of the included studies, while the second part was the quantitative synthesis of the evidence in terms of available categorical or continuous data for pooling by statistical method. Qualitative synthesis of the risk or odds of malaria infection in patients with BLL was performed as the outcome of each included study could not be pooled. The pooled mean BLL and prevalence of malaria infection in the included studies were estimated using a random-effect model. The heterogeneity of the outcomes among the included studies was assessed using the Cochran Q test and I^2^ statistics. The subgroup analysis of study sites and participants was performed to explore the source(s) of heterogeneity of the outcomes. Publication bias was assessed in the case of more than 10 studies used for pooling the same outcome. All analyses were performed using Stata version 14 (StataCorp, College Station, TX, USA).

## 3. Results

### 3.1. Search Results

**Figure 1:**
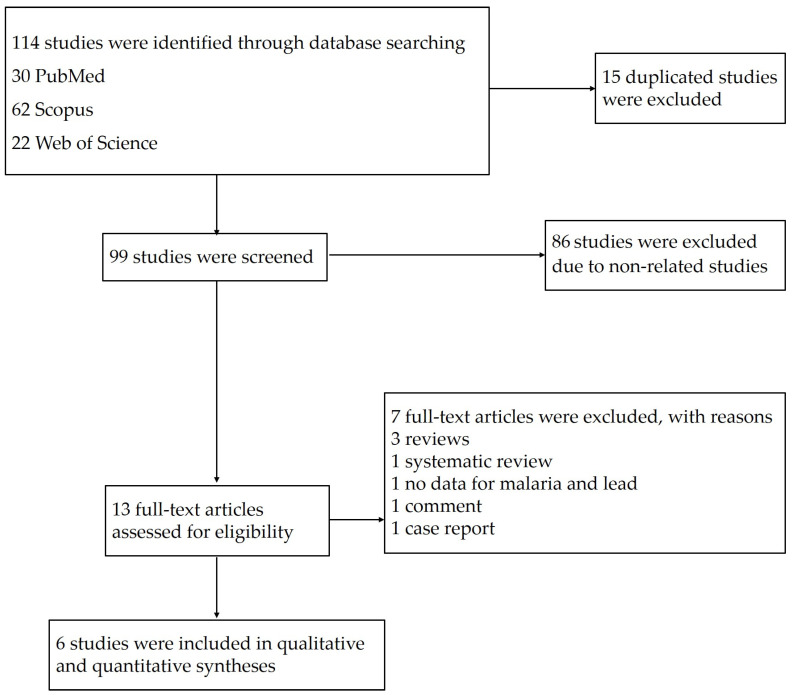
Study flow diagram of the study selection.

The searches in the three databases identified 114 potentially relevant studies. After 15 duplicates were removed, the titles and abstracts of 99 studies were examined; then, 86 studies were excluded as non-related studies. The remaining 13 studies were assessed for full texts; then, seven studies were excluded with reasons: three reviews, one systematic review, one study without data on malaria and lead, one comment, and one case report. Finally, six eligible studies [11,18,19,20,21,24] were included in the qualitative and quantitative syntheses (Figure 1).

### 3.2. Characteristics of the Included Studies

Characteristics of the included studies are demonstrated in Table 1. The included studies were published between 2011 and 2020 and conducted between 2005 and 2013. Three studies were conducted in Nigeria (3/6, 50%) [11,19,20], two studies were conducted in Benin (2/6, 33.3%) [18,24], and one study was conducted in Uganda [21]. Three studies had cross-sectional design [18,19,21], while two studies were prospective observational design [11,20], and one study had a cohort design [24]. Five studies enrolled children age between 1 and 9 years [18,19,20,21,24], while only one study enrolled pregnant women [11]. Among studies that enrolled children, one study enrolled children with venous blood lead level (VBLL) >45 mg/dL before chelation [20], and one study enrolled children with malaria [21]. Four studies aimed to investigate the correlation between malaria and BLL [11,18,19,24], while two studies aimed to determine the impact of both malaria and BLL on patients’ complications, such as neurological complications [20] and severe anemia [21]. Three studies [11,18,21] used microscopy for malaria diagnosis, while others used both microscopy or rapid diagnostic test (RDT) [24], RDT alone [20], or did not specified the test method [19]. For BLL assessment, mass spectrometry [18,20,21,24], inductively coupled plasma/mass spectrometry [19], and atomic absorption spectrophotometry [11] were used. Regarding the quality of the included studies, three studies had high quality [11,18,24] while the other three studies had moderate quality [19,20,21].

### 3.3. Mean BLL of Participants Enrolled in the Included Studies

**Figure 2:**
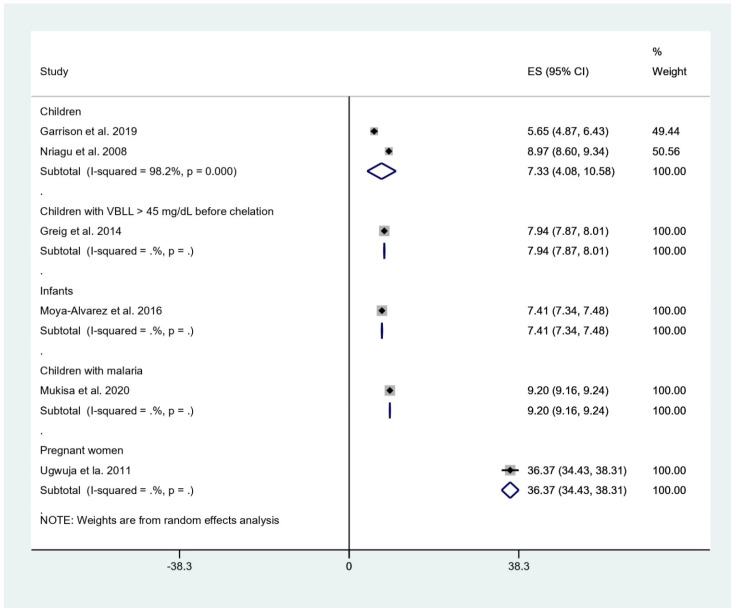
Mean BLL of participants. ES, effect size (mean BLL in μg/dL); CI, confidence interval (μg/dL); black diamond symbol, point estimate; solid line in the middle of the graph at 0, zero effect size.

The pooled mean BLL was estimated and stratified to five subgroups, including children, children with BLL > 45 mg/dL before chelation, infants, children with malaria, and pregnant women. The result showed that the pooled mean BLLs were 7.33 μg/dL in children (95% confidence interval [95%CI], 4.08–10.58, I^2^: 98.2%), 7.94 μg/dL in children with BLL > 45 mg/dL before chelation (95%CI, 7.87–8.01), 7.41 μg/dL in infants (95%CI, 7.34–7.48 μg/dL), 9.20 μg/dL in children with malaria (95%CI, 9.16–9.24 μg/dL), and 36.37 μg/dL in pregnant women (95%CI, 34.43–38.31 μg/dL) (Figure 2).

### 3.4. Prevalence of Malaria among Participants

**Figure 3:**
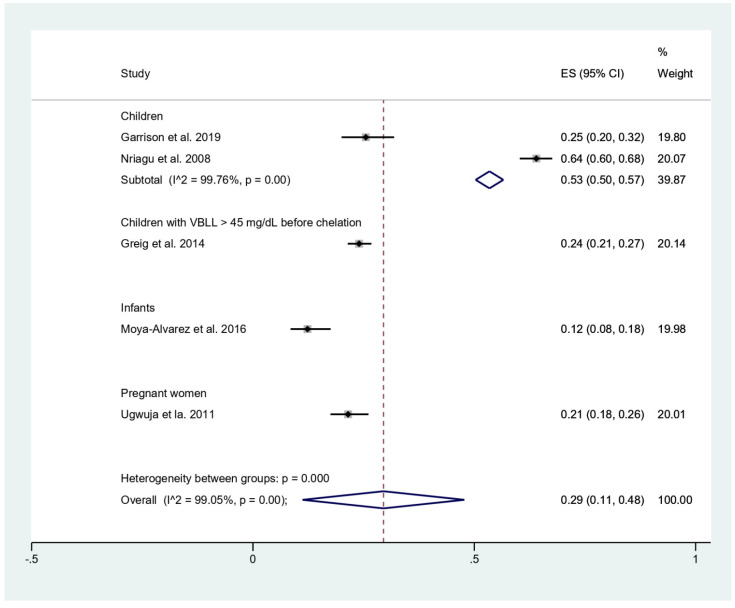
Pooled prevalence of malaria by types of participants. ES, effect size (prevalence of malaria); CI, confidence interval; black diamond symbol, point estimate; solid line in the middle of the graph at 0, zero effect size.

**Figure 4:**
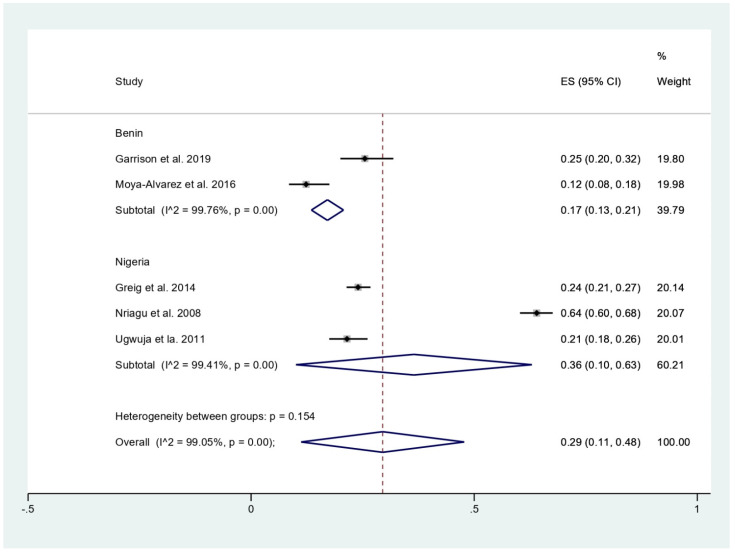
Pooled prevalence of malaria among participants by country. ES, effect size (prevalence of malaria); CI, confidence interval; black diamond symbol, point estimate; solid line in the middle of the graph at 0, zero effect size.

The prevalence of malaria among participants (2381 participants) was estimated using the data from fives studies that reported 803 malaria-positive patients [11,18,19,20,24]. The results showed that the prevalence rates of malaria were 53% in children (95%CI, 50–57%; I^2^, 99.8%), 24% in children with BLL > 45 mg/dL before chelation (95%CI, 21–27%), 12% in infants (95%CI, 8–18%), and 21% in pregnant women (95%CI, 18–26%) (Figure 3). Subgroup analysis of countries demonstrated that the prevalence of malaria among participants was 17% in Benin (95%CI, 13–21%; I^2^, 98.8%), and 36% in Nigeria (95%CI, 10–63%; I^2^, 99.4%) (Figure 4). Overall, the pooled prevalence rate of malaria infection among participants was 29% (95%CI, 11–48%; I^2^, 99.1%).

### 3.5. BLL and Risk of Malaria Infection

BLL and risk of malaria infection were analyzed by five studies [11,18,19,21,24]. BLL associated with decreased risk of malaria was demonstrated by two studies conducted in Benin [18] and Nigeria [19], while BLL associated with increased risk of malaria was demonstrated by a study that was conducted in Nigeria [11]. No association between BLL and malaria or BLL and malaria parasite density was demonstrated in studies conducted in Benin [24] and Uganda [21], respectively.

### 3.6. BLL and Risk of Severe Malaria

BLL and risk of severe malaria were demonstrated by two studies conducted in Nigeria [20] and Uganda [21]. The study conducted in Nigeria that enrolled children with BLL ≥ 45 µg/dL before any chelation showed a positive correlation between severe neurological features in patients with malaria. Moreover, severe neurological features were observed in children with a positive malaria RDT at VBLL as low as 50 mg/dL [20]. In the study conducted in Uganda [21], no association between BLL and malaria parasite density was observed (*p*, 0.082; correlation values r, 0.124). However, there was a higher mean parasite density in severely anemic patients with malaria (7400 parasites/μL) compared to that in those without anemia (1700 parasites/μL). They also showed a positive correlation between BLL and hemoglobin level (*p* < 0.001; correlation values r, 0.552) and a negative correlation for malaria parasite density and hemoglobin level (*p*, 0.035; correlation values r, −0.231) [21].

## 4. Discussion

The systematic review and meta-analysis showed that the pooled mean BLL among children who were investigated for malaria was approximately 7.33 μg/dL with a high heterogeneity among the two studies (I^2^, 98.2%) [19,24]. The source of heterogeneity might be the difference in study site, as one was conducted in Benin [24] and the other study was conducted in Nigeria [19], or the difference in the study design, as one was a cohort study [24], while another was a cross-sectional study [19]. Among infants, a higher mean BLL of 7.41 μg/dL was observed [18], and sources of lead contamination might have been paint, piped water, leaded gasoline, or consumption of animals killed by ammunition. Moreover, high BLL in infants might be caused by iron deficiency, which leads to increased lead absorption [24]. Furthermore, in children with specific conditions, such as BLL > 45 mg/dL before chelation or positive malaria test, a higher mean of BLL was observed at 7.94 and 9.20 μg/dL, respectively. Interestingly, the highest mean BLL of 36.37 μg/dL was demonstrated in pregnant women who received antenatal care at the hospitals [11]. The high mean BLL in pregnant women might be caused by the high sensitivity of pregnant women to lead exposure due to biological changes [25]. 

The present meta-analysis showed that the prevalence rate of malaria was highest in children (53%), while a lower prevalence rate was demonstrated in children with BLL > 45 mg/dL before chelation (24%), pregnant women (21%), and infants (12%). Among children, a high heterogeneity of the prevalence (I^2^, 99.8%) was observed. This heterogeneity might be also caused by the difference in study designs or sites. The subgroup analysis of study sites showed that the prevalence rate of malaria was high in Nigeria (36%) and lower in Benin (17%). However, heterogeneity of the prevalence existed. Therefore, the difference in the prevalence might be due to other factors, such as geographical distribution of malaria parasites in the African region. According to the WHO report in 2020, Nigeria was the country with the highest malaria burden share (27%), while the malaria burden share in Benin was only 2% [17]. Nevertheless, the prevalence of malaria in these two countries is quite similar based on the demographic and health surveys in 2017–2018 in children aged <5 years: between 36% and 39% in Benin and 36% and 22% in Nigeria with RDT and microscopy, respectively [17].

For BLL and risk of malaria infection, previous studies in Benin [18] and Nigeria [19] showed that BLL was associated with decreased risk of malaria. High BLL was associated with a reduction in malaria risk as it reduced the possibility of a positive blood smear or reduced malaria parasite density [18]. Therefore, high BLL might have the advantage of protection against malaria infection in infants as they have a high risk of mortality. Nevertheless, the high BLL in infants raises concern about the possible harmful consequences for the infant’s health rather than the protective effect for malaria. In contrast to the lead protection against malaria theory, another study conducted in pregnant women in Nigeria showed that BLL was associated with increased risk of malaria infection [11]. The mechanism by which lead might protect against or influence malaria infection has not been elucidated so far. There were some possible explanations of lead protection against malaria infection; for example, lead can modulate the host immune response during malaria infection [26,27] and, in turn, alter heme synthesis or iron metabolism, which interferes with the iron utilization of malaria parasites [28]; influence the Th1/Th2 balance [29,30]; or inhibit protein synthesis [31]. Furthermore, lead-induced iron deficiency can influence the antiparasitic effect [28]. Although lead exposure can increase or decrease the susceptibility to malaria infection, some studies that were conducted in Benin [24] and Uganda [21] reported no association between BLL and risk of malaria or malaria parasite density. The absence of association between BLL and malaria in the study that was conducted in Benin [24] might be because children received iron supplements to treat iron-deficiency anemia during the study period or because children with malaria were subsequently treated within the study period. Another study suggested that BLL had little or no direct effect on malaria parasite density; however, the study did not find a direct relationship between high BLL and parasite density, and the association between BLL and malaria should be further investigated by prospective observational studies or cohort studies.

The association between BLL and malaria severity was also demonstrated by two studies conducted in Nigeria [20] and Uganda [21]. The study that was conducted in Nigeria [20] demonstrated that severe neurological features in children were associated with positive malaria at BLL as low as 50 mg/dL compared to those in children without malaria at 105 mg/dL. Therefore, the neurological features of children who live in the malaria-endemic area are needed to distinguish from lead encephalopathy. The mechanism of malaria-induced hemolysis can increase the BLL, which, in turn, increases the lead available to cause encephalopathy [20]. Moreover, lead poisoning might protect against severe malaria by altering the immune system [32]. The study in Uganda suggested that the BLL and malaria infection could drive the progression of patients to severe anemia due to red blood cell destruction, ineffective erythropoiesis, and interference of the hepcidin iron regulatory system [21]. However, these studies did not assess the nutritional status of the participants, which might be involved in the null association between BLL and malaria.

The main limitation of the present systematic review and meta-analysis was the limited number of studies that assessed the BLL and malaria; therefore, the meta-analysis on risk or odds of malaria infection influenced by BLL could not be determined. Furthermore, a high heterogeneity of the outcomes, such as pooled mean BLL and pooled prevalence of malaria infection, among participants was observed; hence, the result of the individual study or subgroup of studies should be interpreted. In conclusion, the present systematic review and meta-analysis determined the current status of the studies on BLL and risk of malaria in African countries. Moreover, the heterogeneity of the association between BLL and malaria infection was observed. Further studies are needed to investigate the impact of BLL on patients with malaria to help the clinician determine the risk of severity, such as the development of neurological features and severe anemia, in patients exposed to lead.

## Figures and Tables

**Table 1:** Characteristics of the included studies.

Author	Study Site	Year of Conduction	Study Design	Number of Participants	Participants	Age Range (Years)	Male %	Research Questions	Mean ± SD BLL (μg/dL)	Number of Malaria	Outcomes	Outcome Parameters	Test for Malaria	Test for BLL
Garrison et al., 2019	Benin	2008–2013	Cohort study	204	Children	1–2	51	BLL and malaria	5.65 ± 5.683	52	No association between BLL and malaria	IRR of BLL in 4th quartile and malaria, 0.94 (95%CI, 0.68, 1.30, *p* = 0.7)	Microscopy, RDT	Mass spectrometry
Greig et al., 2014	Nigeria	2010–2011	Prospective observational study	972	Children with VBLL > 45 mg/dL before chelation	<5	49.02	Effect of BLL on neurological features in malaria and non-malaria	7.94 ± 1.115	233	Malaria associated with BLL < 80 mg/dL and any neurological features	OR of malaria and neurological features (*p* = 0.016)	RDT	Mass spectrometry
Moya-Alvarez et al., 2016	Benin	2010–2012	Cross-sectional study	203	Infants	1	48.48	BLL and malaria	7.41 ± 0.514	25	BLL associated with decreased risk of malaria	AOR of BLL in 4th quartile and malaria, 0.19 (95%CI, 0.04–0.95, *p* = 0.04)	Microscopy	Mass spectrometry
Mukisa et al., 2020	Uganda	NS	Cross-sectional study	198	Children with malaria positive	NS	NS	BLL and anemia among malaria	9.2 ± 0.3	198	No association between BLL and malaria parasite density	Correlation coefficient of BLL and parasite density, 0.124 (*p* = 0.082)	Microscopy	Mass spectrometry
Nriagu et al., 2008	Nigeria	2005–2006	Cross-sectional study	653	Children	2–9	56.5	BLL and malaria	8.97 ± 4.8	418	BLL associated with decreased risk of malaria	Correlation coefficient of BLL and malaria, −0.149 (*p* < 0.0001)	NS	Inductively coupled plasma/mass spectrometer (ICP–MS)
Ugwuja et al., 2011	Nigeria	2007–2008	Prospective observational study	349	Pregnant women	15–40	0	BLL and pregnancy outcomes	36.37 ± 18.45	75	BLL associated with increased risk of malaria	OR of BLL and malaria, (*p* < 0.05)	Microscopy	Atomic absorption spectrophotometer

IRR, incidence rate ratio; BLL, blood lead level; OR, odds ratio; AOR, adjusted odds ratio; NS, not specified.

## Data Availability

All data relating to the present study in this manuscript are available.

## Supplementary Materials

The following are available online at https://www.mdpi.com/article/10.3390/tropicalmed6030149/s1, Table S1. Search term, Table S2. Quality of the included studies, PRISMA Checklist S1.
