# Supplementary material for: Blood Lead Levels and Subsequence Risk of Malaria in the African Population: A Systematic Review and Meta-Analysis
Source: Trop Med Infect Dis. 2021 Aug 8;6(3):149. doi: 10.3390/tropicalmed6030149 (PMC8396233; doi:10.3390/tropicalmed6030149)
Supplement: Supplementary file 1 [file tropicalmed-06-00149-s001.zip › Table S1. Search term.pdf]

Blood lead levels and subsequence risk of malaria in the African population: a systematic review and meta-analysis

Saruda Kuraeiad[1], Manas Kotepui [1*]

[1] Medical Technology, School of Allied Health Sciences, Walailak University, Tha Sala, Nakhon Si Thammarat, Thailand

Authors' Email Addresses:

[*]**Corresponding Author**: Manas Kotepui; manas.ko@wu.ac.th

Saruda Kuraeiad; saruda.ku@wu.ac.th

Table S1. Search term

Databases	Search terms	Search date
MEDLINE	("lead exposure" OR "lead toxicity" OR "blood lead" OR "lead level" OR "lead pollution" OR "exposure to lead" OR "lead level" OR "lead poisoning" OR "lead ions") AND (malaria OR plasmodium)	20 May 2021
Scopus	("lead exposure" OR "lead toxicity" OR "blood lead" OR "lead level" OR "lead pollution" OR "exposure to lead" OR "lead level" OR "lead poisoning" OR "lead ions") AND (malaria OR plasmodium)<br><br>Search option: All fields	20 May 2021
ISI Web of Science	("lead exposure" OR "lead toxicity" OR "blood lead" OR "lead level" OR "lead pollution" OR "exposure to	20 May 2021

	lead" OR "lead level" OR "lead poisoning" OR "lead ions") AND (malaria OR plasmodium)<br><br>Search option: All fields	
