# Supplementary material for: Blood Lead Levels and Subsequence Risk of Malaria in the African Population: A Systematic Review and Meta-Analysis
Source: Trop Med Infect Dis. 2021 Aug 8;6(3):149. doi: 10.3390/tropicalmed6030149 (PMC8396233; doi:10.3390/tropicalmed6030149)
Supplement: Supplementary file 1 [file tropicalmed-06-00149-s001.zip › Table S2. Quality of the included studies.pdf]

Blood lead levels and subsequence risk of malaria in the African population: a systematic review and meta-analysis

Saruda Kuraeiad[1], Manas Kotepui [1*]

[1] Medical Technology, School of Allied Health Sciences, Walailak University, Tha Sala, Nakhon Si Thammarat, Thailand

Authors' Email Addresses:

[*]**Corresponding Author**: Manas Kotepui; manas.ko@wu.ac.th

Saruda Kuraeiad; saruda.ku@wu.ac.th

No.	Authors	Eligibility criteria	Study subjects and the setting	Exposure measured in a valid and reliable way 'gold standard'	A specified diagnosis or definition	Confounding factors	Dealing with confounding factors	Outcomes measured in a valid and reliable way	Appropriate statistical analysis	Scores (8)	Quality (high, moderate, low)
1	Garrison et al., 2019	Yes	Yes	Yes	Yes	No	NA	Yes	Yes	7	High
2	Greig et al., 2014	Yes	Yes	No	Yes	No	NA	Yes	Yes	6	Moderate
3	Moya-Alvarez et al., 2016	Yes	Yes	Yes	Yes	No	NA	Yes	Yes	7	High
4	Mukisa et al., 2020	Yes	No	Yes	Yes	No	NA	Yes	Yes	6	Moderate
5	Nriagu et al., 2008	Yes	Yes	No	Yes	No	NA	Yes	Yes	6	Moderate
6	Ugwuja et la., 2011	Yes	Yes	Yes	Yes	No	NA	Yes	Yes	7	High

NA, Not Applicable
